# Complex polymeric nanomicelles co-delivering doxorubicin and dimethoxycurcumin for cancer chemotherapy

**DOI:** 10.1080/10717544.2022.2073403

**Published:** 2022-05-25

**Authors:** Muhammad Sohail, Bin Yu, Zheng Sun, Jiali Liu, Yanli Li, Feng Zhao, Daquan Chen, Xin Yang, Hui Xu

**Affiliations:** a School of Pharmacy, Collaborative Innovation Center of Advanced Drug Delivery System and Biotech Drugs, Universities of Shandong, Key Laboratory of Molecular Pharmacology and Drug Evaluation (Yantai University), Ministry of Education, Yantai University, Yantai, China; b School of Chemistry and Chemical Engineering, Yantai University, Yantai, China

**Keywords:** Complex polymeric nanomicelles, doxorubicin, dimethoxycurcumin, combinational cancer chemotherapy

## Abstract

Combinational therapy is a new trend in medical sciences to achieve a maximum therapeutic response of the drugs with a comparatively low incidence of severe adverse effects. To overcome the challenges of conventional formulations for cancer chemotherapy, a polymer-based complex nanomicellar system, namely CPM-DD, was developed co-delivering the anti-cancer agent doxorubicin (DOX) and potent antioxidant dimethoxycurcumin (DiMC). The optimal mass ratio of DOX/DiMC in CPM-DD was determined as 1:6 due to the synergistic antiproliferative effect from *in vitro* cytotoxicity assay, while the biocompatible diblock copolymer of mPEG2000-PLA5000 was selected for drug entrapment at an optimal feeding ratio of 9:1 to both drugs together. The uniform particles of CPM-DD with suitable particle size (∼30 nm) and stable drug loading content (>9%) could be reliably obtained by self-assembly with the encapsulation yield up to 95%. Molecular dynamics simulation revealed the interaction mechanism responsible for forming these complex nanomicelles. The acid-base interaction between two drugs would significantly improve their binding with the copolymer, thus leading to good colloidal stability and controlled drug release characteristics of CPM-DD. Systematic evaluation based on the MCF-7 breast tumor-bearing nude mice model further demonstrated the characteristics of tissue biodistribution of both drugs delivered by CPM-DD, which were closely related to the drug loading pattern and greatly responsible for the improved anti-cancer potency and attenuated toxicity of this complex formulation. Therefore, all the findings indicated that CPM-DD would be a good alternative to the conventional formulations of DOX and worthy of clinical application for cancer chemotherapy.

## 1. Introduction

Cancer is a kind of disease with the most remarkable fatality rate globally and the most challenging hurdle to overcome in aging humans (Bray et al., 2018). Chemotherapeutic agents clinically still play a significant role in eradicating tumor cells because of their efficacies, among which the anthracycline antibiotic doxorubicin (DOX) is a common component of multiple chemotherapy drug regimens used to treat various solid and hematological malignancies such as breast carcinoma, soft tissue sarcomas, osteosarcoma, leukemia and lymphomas (Moraes et al., 2021; Sohail et al., 2021a; Waks & Winer 2019). There are several molecular targets involved in the cytotoxicity of DOX, and its substantial anti-cancer action could be ascribed to DNA intercalation and generation of ROS and centered-carbon radicals (Tacar et al., 2012; Gonçalves et al., 2020). However, the clinical use of DOX as a chemotherapeutic drug is frequently linked to a variety of dose-limiting side effects, especially the risk of cardiac problems such as dilated cardiomyopathy, congestive heart failure, and early mortality, which may impact up to 11% of patients and remains a huge challenge (Chatterjee et al., 2010; Zhang et al., 2018).

Dimethoxycurcumin (DiMC) is a semi-synthetic derivative of curcumin with much-improved bioactivities such as anti-inflammation (Patwardhan et al., 2011) and antioxidant/pro-oxidant effects (Liu et al., 2015). More to the point, it could display much higher antiproliferative activity as compared to curcumin (Zanetti et al., 2021). It has been demonstrated that the tremendous therapeutic potential of DiMC is due to resistance and stability to metabolic processes (Tamvakopoulos et al., 2007; Sohail et al., 2021b). DiMC thus is regarded as a promising anti-cancer agent with superior metabolic stability against various human cancers, along with good biosafety for normal cells/tissues (Kunwar et al., 2012; Yoon et al., 2014; Hassan et al., 2016; Zhao et al., 2017; Zanetti et al., 2019). Nevertheless, research regarding the drug delivery system on DiMC is currently quite limited (Liu et al., 2015). It has become challenging to actualize further development and clinical use of the multi-target therapeutic potential for cancer.

There are several drawbacks of systemic administration of a single chemotherapeutic, and the combination therapy is sequentially developed to overcome multi-drug resistance and side effects because of using a lower concentration of each drug (Zhang et al., 2016; Bayat et al., 2017; Ramasamy et al 2017). Currently, a variety of nanotechnologies have been extensively applied to improve delivery systems for cancer treatment (Zhou et al., 2020; Li et al., 2021a; Li et al., 2021b; Sohail et al., 2021c; Zhang et al., 2022). Co-loading of multiple anti-cancer drugs into a single nanocarrier system provides a logical approach to combination therapy, and various collections of nanocarriers are included, for example, liposomes, dendrimers, nanoparticles, micelles etc. (Duan et al., 2013; Meric-Bernstam et al., 2021; Fan et al., 2017; Chen et al., 2017; Elzoghby et al., 2016; Luo et al., 2017). Based on the abnormal vasculature of the tumor, these nanostructures usually accumulate in tumors through the enhanced permeability and retention (EPR) effect (Han et al., 2020; Lin et al., 2021). Particularly the polymeric micelles have gained much interest due to the unique self-assembling nature of amphiphilic copolymers (Peng et al., 2008; Ma et al., 2017; He et al., 2020). More importantly, polymeric micelles usually have a uniform particle size, high thermal stability, biocompatibility, and the capability of encapsulating both hydrophilic and hydrophobic drugs (Tan et al., 2017; Huang et al. 2017).

**Figure 1.**
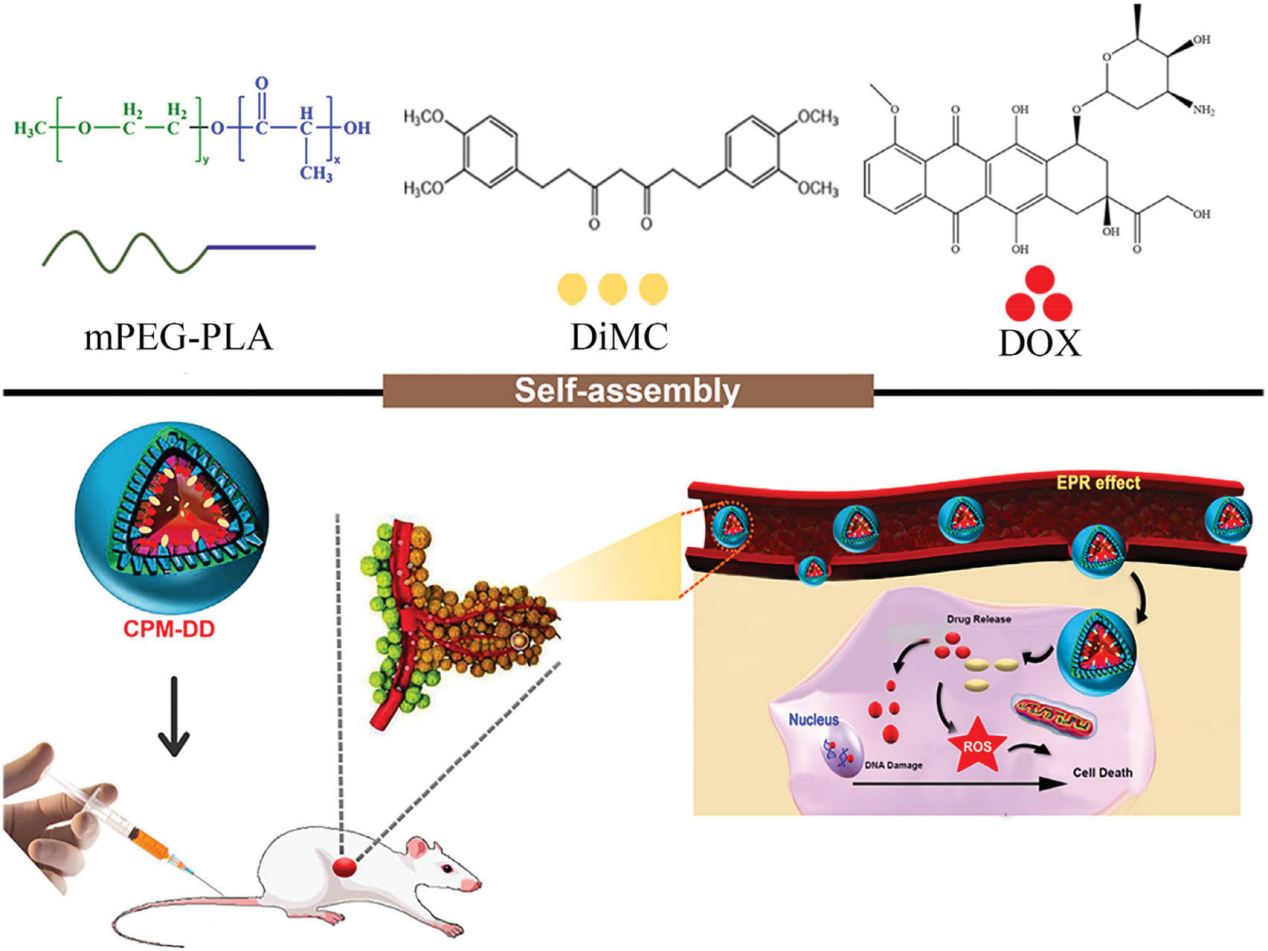
Schematic representation of the self-assembly complex nanomicelles of CPM-DD for tumor targeting therapy.

The present study aimed to develop CPM-DD, a kind of complex polymeric nanomicellar system co-delivering DOX and DiMC, the two anti-cancer agents with different mechanisms of action for combination chemotherapy (Figure 1). The amphiphilic diblock copolymer of monomethoxy poly(ethylene glycol) (mPEG)-poly(lactic acid) (PLA), mPEG-PLA has been approved by the FDA was used as a drug carrier. Both the formulation and process conditions were optimized as per the demand of the standard clinical application of DOX-related chemotherapy via nanomicelles, mainly including high drug loading capacity, good colloidal stability, and tissue-targeting feature (Li et al., 2020; Sohail et al, 2021b; 2021c). After a systemic characterization, the mechanism of drugs encapsulation into CPM-DD was investigated by molecular dynamic simulation, and the therapeutic potential was evaluated by a set of tests *in vitro* and *in vivo*. To the best of our knowledge, there have been no reports of complex nano-formulation of DOX and DiMC. We expected that the resulting complex polymeric micellar system CPM-DD would provide a good alternative to overcome the main drawbacks of DOX-based cancer chemotherapies.

## 2. Materials and methods

### 2.1. Materials

DOX (doxorubicin hydrochloride) was obtained from Beijing Ouhe Technology Co., Ltd (Beijing, China), and dimethoxycurcumin (DiMC) was laboratory-made with an HPLC purity >98%. The copolymers of mPEG-PLA with various block length ratios (mPEG1000-PLA2000, mPEG2000-PLA2000, mPEG2000-PLA5000) were supplied by Shanghai Leon Chemical Ltd (Shanghai China). The dialysis tubes with a molecular mass cutoff of 8–14 KDa were purchased from Spectrum Laboratories (Houston, USA). The Cell Counting Kit-8 (CCK-8) was the product of Dalian Meilun Biotech Co., Ltd (Dalian, China), and the kits for biochemical assay were supplied by Nanjing Jiancheng Biochemistry Co. Ltd. (Nanjing, China). All solvents and other reagents were available commercially and of analytical grade or higher. Ultra-pure water prepared by a lab purification system was used throughout the experiment.

### 2.2. Cells and animals

All the human cancer cell lines such as human lung cancer cell line A549, human liver cancer cell line SMMC-7721, human colon cancer cell line HT-29, and human breast cancer cell line MCF-7 were obtained from the Cell Bank of the Chinese Academy of Sciences (Shanghai, China). DMEM (10% fetal bovine serum, 1% penicillin/streptomycin, HyClone Laboratories, Logan, USA) was used for cell culture in a humidified incubator with 5% CO_2_ at 37 °C. Female BALB/c nude mice (4–5 weeks old, weighing 20 ± 2 g) were purchased from the Shanghai Experimental Animal Center of the Chinese Academy of Sciences (Shanghai, China). All experimental animal procedures were conducted following the National Institutes of Health (NIH) guidelines and approved by the Animal Experimentation Ethics Committee of Yantai University, China.

### 2.3. Preparation of CPM-DD

By using the amphiphilic diblock copolymer mPEG-PLA as a drug carrier, the nanomicelles CPM-DD co-encapsulating DOX and DiMC were prepared via the classic thin-film hydration method according to a patent two-step way as previously reported (Zhang et al., 2018). In brief, a specified amount of copolymer and DiMC were dissolved in anhydrous acetone at first. After 5 minutes of stirring, the solvent was slowly evaporated under a water bath (45 ± 2 °C) to form a thin-layer film, dissolved with physiological saline to obtain a transparent micelle solution, followed by successive addition of the phosphate-buffered solution (PBS; 10×, pH 7.4) and the concentrated aqueous solution of DOX. The mixture was stirred at room temperature for about 20 min, and filtered through a 0.22-μm filter to obtain CPM-DD. Then the freeze-dried powder was prepared for storage by a subsequent lyophilization process (FD-1C-80 freeze-dryer, Shanghai, China).

### 2.4. Characterization of CPM-DD

The micelles were reconstituted to obtain an aqueous solution (∼1 mg/mL) for morphology observation by using transmission electron microscopy (JEM-1400 TEM, JEOL, Tokyo, Japan). By the dynamic light scattering (DLS) method, the particle size of micelles was measured, and the polydispersity index (PDI) was determined to evaluate the distribution of particle size (Zetasizer Nano ZS 90, Malvern, UK). Moreover, the colloidal stability of micelles was investigated by measuring the particle size according to the schedule after incubation with fetal bovine serum (FBS, 1% or 10%) in PBS solution (pH 7.4) at 37 °C under gentle stirring.

HPLC-UV quantification assay of both DOX and DiMC in micelles was performed simultaneously via a Waters e2695 HPLC system for determination of drug loading content (DLC) and encapsulation yield (EY) according to the following Equations (1) and (2), as well as assessment of *in vitro* drug release by using a dialysis tube in PBS (pH 7.4, 0.5% polysorbate 80). In brief, the chromatographic separation was conducted on a TC-C18 column (250 mm ×4.6 mm i.d., 5 μm; Agilent Technologies) at 30 °C, and the injection volume was 20 μL. The mobile phase composed of acetonitrile (A) and 0.1% formic acid (B) was delivered at a flow rate of 1 ml/min under the gradient elution program with a linear increase of A from 55% to 65% during 0–5 minutes, then holding at 65% A during 5–15 minutes, when the detection wavelength was set at 254 nm for DOX, and 420 nm for DiMC, respectively (Liu et al., 2015; Zhang et al., 2018). The HPLC method was fully validated according to the guidelines of FDA. The calibration curve showed good linearity over the concentration range of 1.0–100 μg/mL for each drug and the recovery was between 98.5% and 100.2% with RSD less than 1.0%.

(1) $$\text{DLC}\;(\%)=\frac{\text{amount}\;\text{of}\;\text{loaded}\;\text{drugs}\;\text{in}\;\text{micelles}}{\text{amount}\;\text{of}\;\text{drug}-\text{loaded}\;\text{micelles}}\times 100$$

(2) $$\text{EY}\;(\%)=\frac{\text{amount}\;\text{of}\;\text{loaded}\;\text{drugs}\;\text{in}\;\text{micelles}}{\text{the}\;\text{theoretical}\;\text{amount}\;\text{of}\;\text{drug}\;\text{in}\;\text{micelles}}\times 100$$

### 2.5. Molecular dynamics simulation for drug loading

Molecular dynamics simulation was performed to investigate the molecular mechanism of drug entrapment into micelles by using the open-source HyperChem software (Professional 80, Hypercube Inc., Gainesville, USA). At first, the 3-D structure of copolymer or small-molecule drug was theoretically simulated using molecular mechanics (MM) and molecular dynamics (MD) (Zhang et al., 2018). According to the initial structure, a series of geometrical optimization was performed at the MM level via the OPLS method using the steepest descent algorithm until the root mean square gradient was less than 0.10 kcal/(mol•Angstrom). After heating from 0 K to 600 K, the optimized structure then was subject to a series of MD simulations running at 600 K with each runtime of 100 ps to obtain a lower energy minimum, for which the CHARMM27 force field was used and the solvent effect was considered implicitly (Jorgensen et al., 1996; Foloppe & MacKerell 2000). Finally, random docking was performed to examine the interactions between drug and copolymer based on the optimal 3 D structures of the copolymer and small-molecule drug of DOX or DiMC.

### 2.6. Assay of *in vitro* cytotoxicity

CCK-8 assay uses a tetrazolium salt (WST-8) to produce the water-soluble WST-8 formazan by receiving two electrons from viable cells through an electron mediator, 1-Methoxy PMS by NADH and NADPH activity. WST-8 formazan is orange-colored and its amount is dependent on the activity of cellular dehydrogenase, so the WST-8/1-Methoxy PMS system has been widely used to determine cell viability (Ishiyama et al., 1997; Matsuoka et al., 2000; Ma et al., 2021). Herein *in vitro* cytotoxicity against various human cancer cell lines such as A549, HT-29, MCF-7 and SMMC-7721 was investigated by using the CCK-8 assay. Briefly, the logarithmic growth phase cells were prepared as single-cell suspensions and seeded into 96-well plates at a density of 4 × 10^3^ per well for pre-incubation overnight. Then the cells were subjected to different treatment paradigms for 24 h incubation. After aspirating the drug and rinsing the cells three times with PBS (pH 7.4), the viability of the cells was determined as per the kit manual by measuring the absorbance of media with CCK-8 (10%, *v*/*v*) after 6 hours of incubation using a microplate reader set to 450 nm. The cell growth inhibition rate was calculated according to the difference in absorbance between the experimental group and the blank control group. All the results were the average measurement of six replicate wells and were expressed as mean ± SD.

### 2.7. Cellular localization study

Herein the human breast cancer MCF-7 cell line was used for cell uptake study using a previously reported method with slight modification (Qin et al., 2018). In brief, the cells (1 × 10^5^ cells/mL) were inoculated on a glass plate (35 mm × 12 mm) for 24 hours, then a fresh DMEM medium containing drug at a similar DOX concentration of 0.1 μM was added after the spent medium was removed, and the cells were cultured for 2 hours. The media was then removed, and the cells were washed three times with PBS (pH 7.4) and then fixed for 15 minutes in a 4% paraformaldehyde solution. After discarding the fixed liquid, cells were rinsed thrice with PBS and Triton X-100 was added to a final concentration of 0.2%, followed by further incubation in DAPI solution (2 mg/L) for 15 minutes at 37 °C. After removing the dye, the cells were rinsed with methanol and sealed with glycerol. Under a confocal laser scanning microscopy (CLSM, Olympus FV1000, Japan), the fluorescence of the cell sample then was observed at an excitation wavelength of 488 nm and the detection wavelength of 543 nm to examine DOX in the cytoplasm and nucleus, while DAPI was excited at 330 nm to determine the location of the nucleus.

### 2.8. Investigation of *in vivo* responses

In the present study, female BALB/c nude mice bearing human breast cancer MCF-7 cells were employed to evaluate *in vivo* responses of CPM-DD such as anti-cancer efficacy, systemic safety, and biodistribution characteristics as well.

Briefly, the tumor-bearing mouse model was developed through subcutaneous injection of MCF-7 cells at a cell count of about 1 × 10^7^ in the right flank, as previously reported (Zuo et al., 2019). After the tumor volume reached ∼200 mm^3^, the mice were randomly grouped into CPM-DD, Cocktail, Free DOX, Blank micelles, and Control groups, which were i.v. administered once every 2 days for repeated 5 times with CPM-DD micelles, free DOX solution, the cocktail solution of DOX and DiMC, blank micelles with no drug, and the normal vehicle saline, respectively. Each group had eight mice and the dosage was 2.5 mg/kg for DOX and 15 mg/kg for DiMC equivalent (Liu et al., 2015; Zhang et al., 2018).

Before each administration, the body weight and tumor geometrical dimensions such as length (L) and width (W) were recorded for each aminal, and the tumor volume (V) was calculated according to the equation V=(L × W^2^)/2 (Zhang et al., 2018). At 24 h after the last dosing, all the mice were sacrificed, tumors and major tissues were immediately harvested, weighed, and stored at −80 °C for further analysis of drug content. Briefly, each tissue sample was homogenized with physiological saline solution (1:5, *w*/*v*), followed by centrifugation (4 °C, 12,000 rpm × 10 min) to obtain the supernate for solvent extraction. Then an aliquot of homogenate sample (1 mL) was taken into a clean EP tube, added 0.1 mL internal standard (IS) solution, 0.3 mL Na_2_CO_3_ solution (0.02 mol/L) and 2 mL ethyl acetate, mixed for 1 min, centrifugated at 4 °C for 10 min (12,000 rpm). The supernate was taken out, dried under nitrogen gas flow, added 0.1 mL mobile phase solution to dissolve the residue for UPLC-MS/MS quantification assay via an AB Sciex Triple Quad^TM^ 4500 system connected with Shimadzu LC-30AD. Positive electro-spray ionization (ESI) was employed as the ionization source, and the injection volume was 5 μL for chromatographic separation on an ACQUITY UPLC BEH C18 column (50 mm ×2.1 mm, 1.7 μm; WATERS) at 30 °C, gradiently eluted with the mixture of acetonitrile and 0.1% formic acid at a flow rate of 0.2 mL/min. The mass spectrometer was operated by multiple reaction monitoring (MRM) of the transitions *m/z* 544.2/396.8 for DOX, *m/z* 397.3/190.9 for DiMC, and *m/z* 749.4/591.5 for the IS azithromycin, respectively.

For histological examination, the paraffin-embedded sections of the left half heart of mice were sliced (5 μm) and then HE staining for light microscope observations was performed (Zhang et al., 2018). All specimens were analyzed, and the representative images were captured by two pathologists with the blind investigation.

### 2.9. Statistics

All the data were presented as mean ± SD of replicate measurements. The student’s *t*-test was applied to data analysis by using the statistical software package SPSS 20.0 (International Business Machines Corporation, New York, USA). Statistical significance was indicated by *p* < .05 and more statistical significance by *p* < .01.

## 3. Results and discussion

### 3.1. Screening optimal DOX/DiMC ratio in CPM-DD

Herein the two drug molecules involved were DOX and DiMC, a chemotherapeutic agent for various malignancies that can cause cell necrosis and apoptosis by interacting with DNA intercalation and inhibiting RNA transcription, and a promising naturally derived multifunctional anti-cancer agent, respectively (Sohail et al., 2021a; 2021b). It is well-known that combination therapy using drugs with different mechanisms can be synergistic, additive or antagonistic in therapeutic effects, depending greatly on the compatibility ratio of drugs (Pritchard et al., 2013). Therefore, the optimal DOX/DiMC compatibility ratio in the complex nanomicelles of CPM-DD was investigated according to the therapeutic responses based on *in vitro* cytotoxicity testing, which was performed via CCK-8 assay using four kinds of human tumor cell lines such as SMMC-7721, HT-29, MCF-7, and A549.

**Figure 2.**
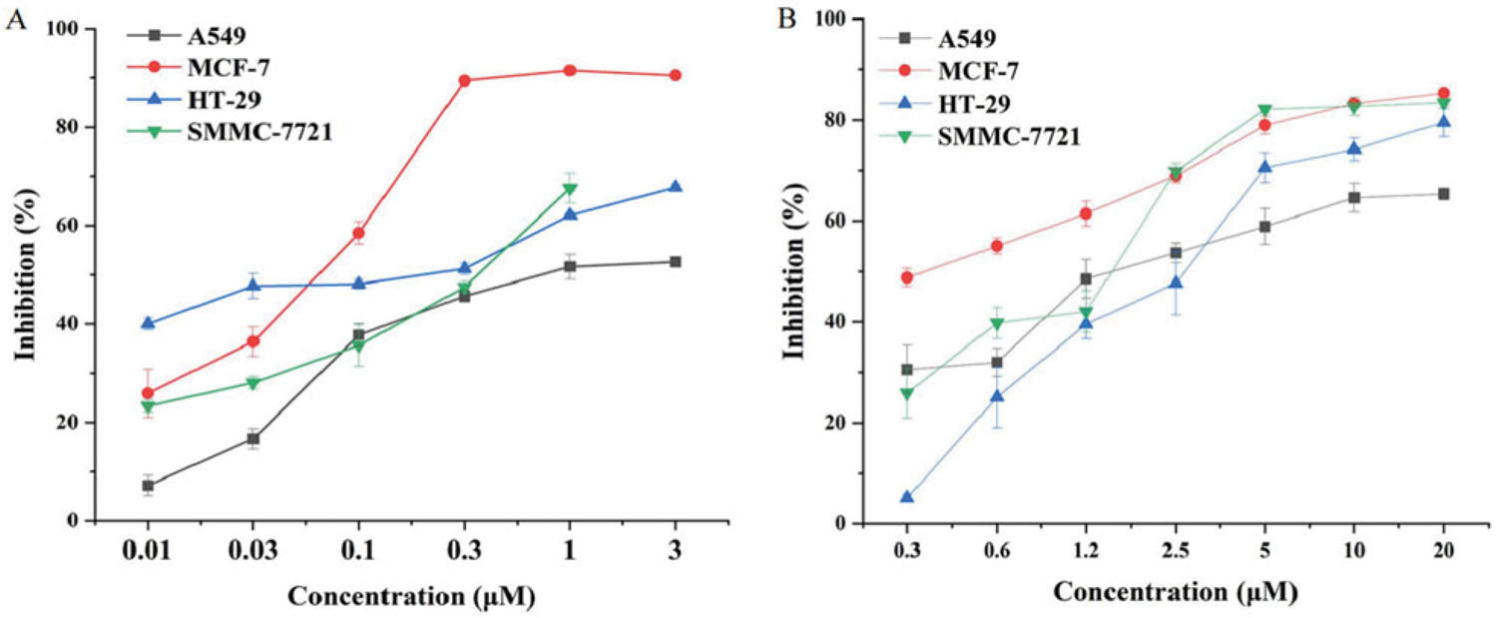
The *in vitro* cytotoxicity of DOX (A) and DiMC (B) against various cancer cell lines.

Resultantly both DOX and DiMC displayed cytotoxicity against *in vitro* growth of the test cell lines, and both clearly exhibited the most potent inhibition against MCF-7 cell proliferation (Figure 2A and B). More to the point, free DOX alone could considerably inhibit *in vitro* proliferation of human breast cancer MCF-7 cells in a concentration-dependent manner with the final drug concentration ranging from 0.01 to 0.3 μM, while that was 0.3 to 10 μM for DiMC. The half inhibitory concentration (IC_50_) against MCF-7 cells was determined to be 0.044 μM (23.9 ng/mL) for DOX, and 0.36 μM (142.7 ng/mL) for DiMC, respectively. These findings demonstrated the strong potency of DOX as a chemotherapeutic agent (Tsou et al., 2015), as well as the obvious difference in cytotoxicity between the two chemicals related to CPM-DD. Thus the MCF-7 cells were chosen for further experiments *in vitro* and *in vivo* to evaluate the combined effects of both drugs.

Under a fixed final DOX concentration of 24 ng/mL close to the IC_50_ value, the inhibition rate of DOX/DiMC combination against *in vitro* growth of MCF-7 cells then was determined with their mass ratio ranging from 1:1 to 1:8. Meanwhile, DOX and DiMC alone at the same final concentration as in combination were tested for comparison. The Q value was further calculated to elucidate the drug combination effect since 0.85 < Q < 1.15 generally indicates an addictive potential, while Q > 1.15 and Q < 0.85 usually stand for a synergistic and antagonistic effect, respectively (Gaibani et al., 2019). Consequently, DOX at 24 ng/mL displayed a mean inhibition rate of 48.9%, and DiMC at 24–192 ng/mL concentration-dependently suppressed cancer cell growth, no matter whether it was used alone or in combination with DOX. Moreover, the calculated Q value ranging from 0.85 to 1.15 was found for each DOX-DiMC combination system except that simultaneously containing 24 ng/mL DOX and 144 ng/mL DiMC, which showed the highest Q value of 1.17 (Table 1). Therefore, the result indicated a synergistic antiproliferative effect of DOX-DiMC combination at a mass ratio of 1:6, but an additive potential for all the other ones. In order to achieve the optimum therapeutic efficacy of CPM-DD with the lowest possible dose of DOX, the drug compatibility of DOX/DiMC was determined at a mass ratio of 1:6 for constructing the complex micelles.

**Table 1.** The inhibitory effect of DiMC alone (I) and DOX-DiMC combination (II) against *in vitro* proliferation of MCF-7 cells (*n* = 3).

Final concentration of DiMC (ng/mL)	Mean inhibition rate/%		*Q* value^b^
	I	II^a^	
24	23.1	53.8	0.886
48	28.3	57.5	0.907
96	40.4	68.1	0.979
144	48.9	86.5	1.171
192	58.2	88.3	1.123

^a^The final DOX concentration was 24 ng/mL for each combination.

^b^The *Q* value was calculated according to the following equation as *E*_(DOX-DiMC)_/(*E*_DOX_+*E*_DiMC_－*E*_DOX_×*E*_DiMC_), where *E*_DOX_ and *E*_DiMC_ meant the inhibition rate caused by DOX or DiMC alone, and *E*_(DOX-DiMC)_ was that of DOX-DiMC combination at the corresponding concentration.

### 3.2. Optimization of formulation and process parameters

Under the optimal drug compatibility ratio of DOX/DiMC, a set of single-factor experiments were further performed for optimizing the formulation and process parameters to build CPM-DD. More to the point, the factors investigated were copolymer type, the feeding ratio of drugs in micelles, and hydration temperature and hydration volume as well.

Amphiphilic copolymers have attracted significant interest as their self-assembly (size and morphology) in an aqueous solution can be precisely modulated (Sohail et al., 2021c). As a kind of diblock copolymer, mPEG-PLA is composed of the most common hydrophilic segment mPEG, and biodegradable hydrophobic material polylactic acid (PLA) approved by the FDA and has been extensively used for preparing polymeric micelles due to good biocompatibility and biodegradation. Some recent studies have demonstrated that mPEG with a molecular weight higher than 3000 might lead to metabolism or other associated immune-genetic problems (Garay et al., 2012; Tan et al., 2017). Therefore, three types of mPEG-PLA copolymer with different molecular weights of the two blocks were investigated for building CPM-DD under the same mass ratio to drugs (9:1), and the key performance indicators such as particle size encapsulation yield were monitored for each system.

As shown in Table 2, mPEG1000-PLA2000 with the shortest hydrophilic chain led to the largest value of mean particle size (260.7 nm) but the lowest encapsulation yield of the micelles (81.2%), which were significantly improved along with the increase of both chains in the copolymer. And consequently, mPEG2000-PLA5000 was found to be the best carrier that would provide the CPM-DD micelles with a moderate particle size (29.7 nm) and optimal manufacturing yield (> 95%) in contrast to the other two copolymers. This result was all consistent with other reports, indicating the great effects of polymer composition on micelle preparation and the performance (Dong & Feng, 2004; Shi et al., 2005).

**Table 2.** Effects of copolymer type and drug feeding ratio on particle size, drug loading content (DLC), and encapsulation yield (EY) of the complex micelles (*n* = 3).

Variable and level		Particle size/nm	DLC/%^a^	EY /%^a^
Copolymer	mPEG_2000_-PLA_5000_	29.7 ± 3.2	9.6 ± 2.5	95.4 ± 9.2
	mPEG_2000_-PLA_2000_	26.4 ± 5.4	8.6 ± 1.2	84.5 ± 12.3
	mPEG_1000_-PLA_2000_	260.7 ± 14.3	6.2 ± 2.3	81.2 ± 6.2
Feeding ratio (drugs/copolymer)	1:7	182.7 ± 6.9	9.2 ± 2.3	71.2 ± 6.2
	1:9	29.5 ± 3.4	9.1 ± 1.5	90.3 ± 5.4
	1:11	42.9 ± 16.3	7.6 ± 1.2	91.1 ± 8.3
	1:13	32.1 ± 5.9	6.5 ± 1.8	90.7 ± 3.4

^a^Both DLC and EY were calculated according to the total amount of two drugs in the complex micelles.

Further taking into account the fact that the micellar preparations in clinical trials usually have the particle size ranging from 20 to 85 nm, and the increase in molecular weight of the polymer carrier may significantly improve the drug pharmacokinetics and tissue distribution, the copolymer mPEG2000-PLA5000 thus was the final choice to build CPM-DD. The CMC value of this copolymer was further determined by using pyrene as a probe via the fluorescence spectroscopy method. The result was about 2.5 mg/L, a fairly low value compared to those commonly used low molecular weight surfactants, which would be responsible for colloidal stability and structural integrity of the micellar system under high dilution conditions *in vivo* (Liu et al., 2015). Meanwhile, the optimal feeding ratio of copolymer to both drugs together was correspondingly determined as 9:1 in the micellar system due to the optimum particle size and encapsulation yield of the aimed micelles (Table 2).

The thin-film hydration method is commonly used for the preparation of various nanocarriers such as polymeric micelles, and the optimum hydration temperature and volume are mainly responsible for micellar drug loading, stability and, shape (Thabet et al., 2022). Taking into account the fact that hydration temperature plays a vital role in the shape of polymeric micelles (Kohori et al., 2002; Akino et al., 2019), the CPM-DD micelles were prepared for evaluation with the hydration temperature ranging from 35 to 65 °C controlled via a water bath. As shown in Table 3, the hydration process at 45 °C provided the micelles with the highest encapsulation yield (>95%) and uniform particles with optimal particle size (about 31 nm), whereas too low or too high hydration temperature led to an obvious decrease in the number of drugs being encapsulated into micelles, along with a significant change in particle sizes.

**Table 3.** Effects of temperature and hydration volume on particle size, drug loading content (DLC), and encapsulation yield (EY) of the complex micelles (*n* = 3).

Variable and level		Particle size/nm	DLC/%^a^	EY/%^a^
Temperature/°C	35	83.2	8.0 ± 3.2	83.4 ± 2.4
	45	31.6	9.6 ± 1.2	95.7 ± 1.4
	55	62.3	8.9 ± 2.3	88.7 ± 1.3
	65	45.5	8.7 ± 4.8	86.4 ± 2.1
Hydration volume/mL	1.5	120.7	6.8 ± 0.3	66.6 ± 2.9
	2.5	24.5	9.6 ± 0.2	94.8 ± 1.3
	3.5	42.9	9.4 ± 0.9	93.5 ± 5.2
	4.5	32.1	9.4 ± 1.8	93.4 ± 3.1

^a^Both DLC and EY were calculated according to the total amount of two drugs in the complex micelles.

Resultantly the final choice was a water bath at 45 °C for hydration. Furthermore, it was found that too small a hydration volume would lead to the inability to hydrate the film completely, thus causing a significant decrease in the encapsulation yield of micelles. The hydration volume up to 2.5 mL was enough to hydrate the drug film completely, and the value ranging from 2.5 to 4.5 mL could provide a fairly stable encapsulation rate and particle size results. Finally, the optimal hydration volume was set as 2.5 mL in order to facilitate the subsequent manufacture of lyophilized powder for storage. All these findings confirmed the influence of hydration factors on the construction of the present complex micellar system.

### 3.3. Characterization of CPM-DD

**Figure 3.**
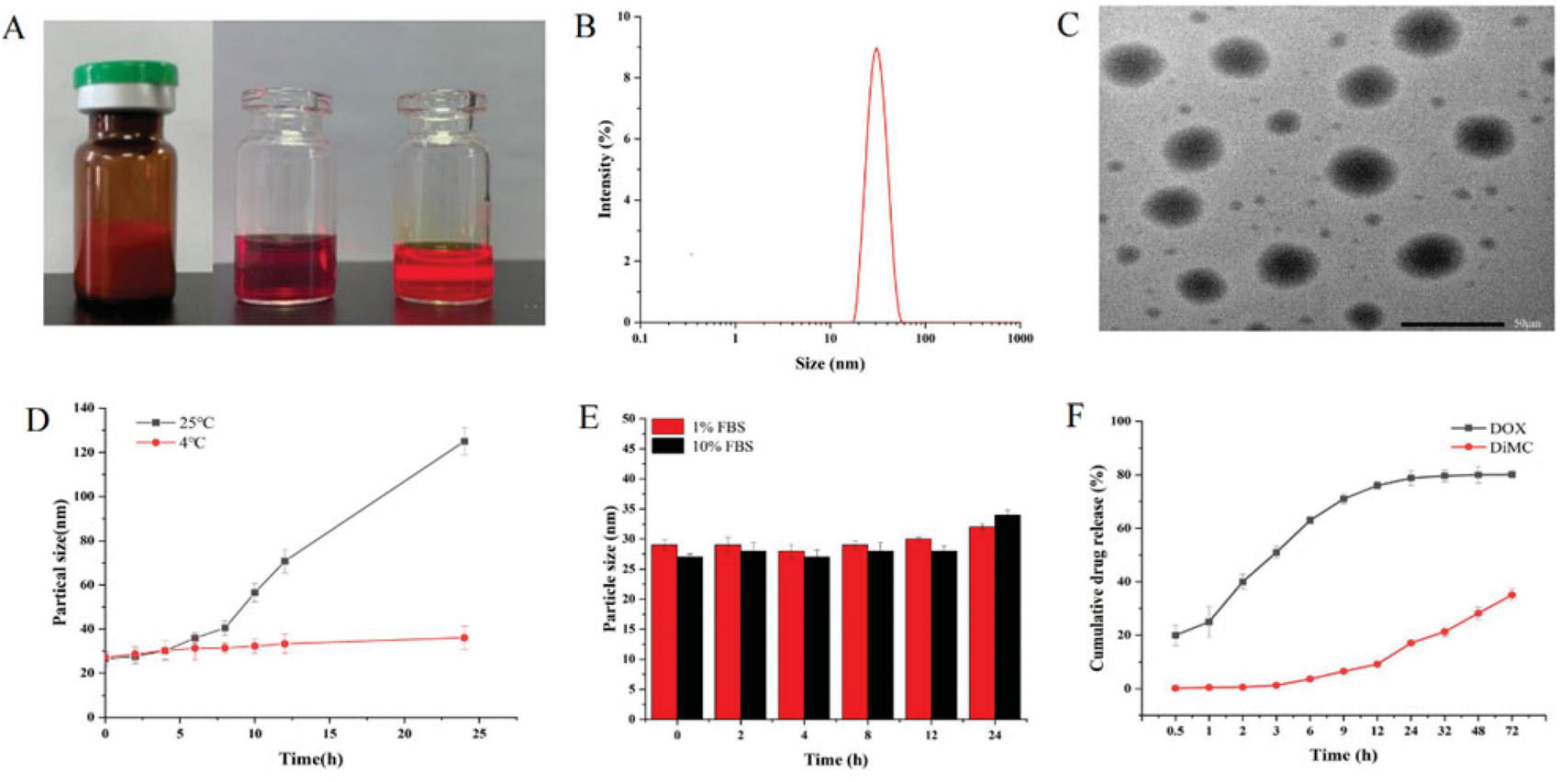
Characterization of CPM-DD. (A) Appearance (→ lyophilized powder, the cocktail solution of both drugs and copolymer, and the freshly prepared micellar solution), (B) Particle size distribution via DLS, (C) TEM imaging, (D) Temperature effect on particle size, (E), FBS effect on particle size, and (F) Drug release profile *in vitro* (pH 7.4).

Based on the optimal formulation and processing conditions, the micelles of CPM-DD were prepared for a systematic characterization. Firstly the lyophilized powder of CPM-DD was found to have an orange-red puffy appearance, and the Tyndall phenomenon could be observed after reconstitution by adding saline (∼2 mg/mL), clearly indicating the colloidal characteristics (Figure 3A). The HPLC analysis demonstrated that the DLC of the two drugs together in micelles was (9.6 ± 0.2) % on average, with the encapsulation yield higher than 95%, and there was no significant difference among batches. DLS assay further revealed that these micelles were small and uniform particles with a mean particle size of about 30 nm and the PDI less than 0.1. Meanwhile, TEM observation clearly illustrated the morphology as spherical particles with a light inner core and a dark outer shell (Figures 3B and C), thus providing the micellar system of CPM-DD with a relatively long circulation in the blood due to the hydrophilic barriers and consequent low drug leakage (Li et al., 2015).

It has been demonstrated that particle size usually has a significant impact on pharmaceutical performance and therapeutic outcomes of nano-drug delivery systems. Therefore, how the particle size changed was monitored under various surrounding conditions to achieve a better understanding of the colloidal stability of CPM-DD. As shown in Figure 3D, the micelles maintained a stable particle size when stored at 4 °C for 24 hours, while a substantial size in the particle could be observed 6 h afterward when stored at 25 °C, suggesting that the lyophilized powder would be an optimal choice to maintain the therapeutic efficacy and pharmaceutical shelf life of CPM-DD.

The micellar particle size was also investigated by using an *in vitro* incubation system of PBS containing FBS at the human body temperature of 37 °C to simulate the real blood circulation system. The results distinctly indicated good stability of the particle dimension within 24 hours when incubated with FBS content ranging from 1% to 10% (Figure 3E). It was interesting to note that CPM-DD in PBS had a slight negative zeta potential of –(1.69 ± 0.15) mV, which got more negative along with the increase of FBS content and reached –(3.1 ± 0.21) mV and –(3.52 ± 0.67) mV in the incubation systems containing 1% and 10% FBS, respectively. These findings thus demonstrated the coating impact of endogenous proteins such as serum albumin on the colloidal stability of CPM-DD micelles.

Finally, *in vitro* drug release properties of CPM-DD were evaluated by utilizing the classic dialysis method, and a PBS solution (pH 7.4) containing 1.5% Tween-80 was used as the release medium to retain the sink condition for both drugs. As a result, DOX and DiMC in the complex polymeric micelles of CPM-DD both displayed a sustained release profile, although there was an obvious difference in the cumulative release rate of the two encapsulated drugs (Figure 3F). More to the point, DOX showed a little higher release (nearly 20%) within the first 0.5 hours, followed by a notably stable release behavior after 1 hour, and reached a cumulative release amount of nearly 84% in 72 hours. Whereas DiMC showed an extended-release profile and reached a total release of 40% within 72 hours, suggesting strong attachments with the copolymer. Such differentiation may be ascribed to the two chemicals encapsulated into micelles' different structures and lipo-hydrophilic character. Furthermore, the Higuchi model could be able to fit the release kinetics well for both drugs, which indicated the kinetics of drug release from CPM-DD was mainly controlled by diffusion mechanisms.

### 3.4. Molecular mechanism of drug entrapment

**Figure 4.**
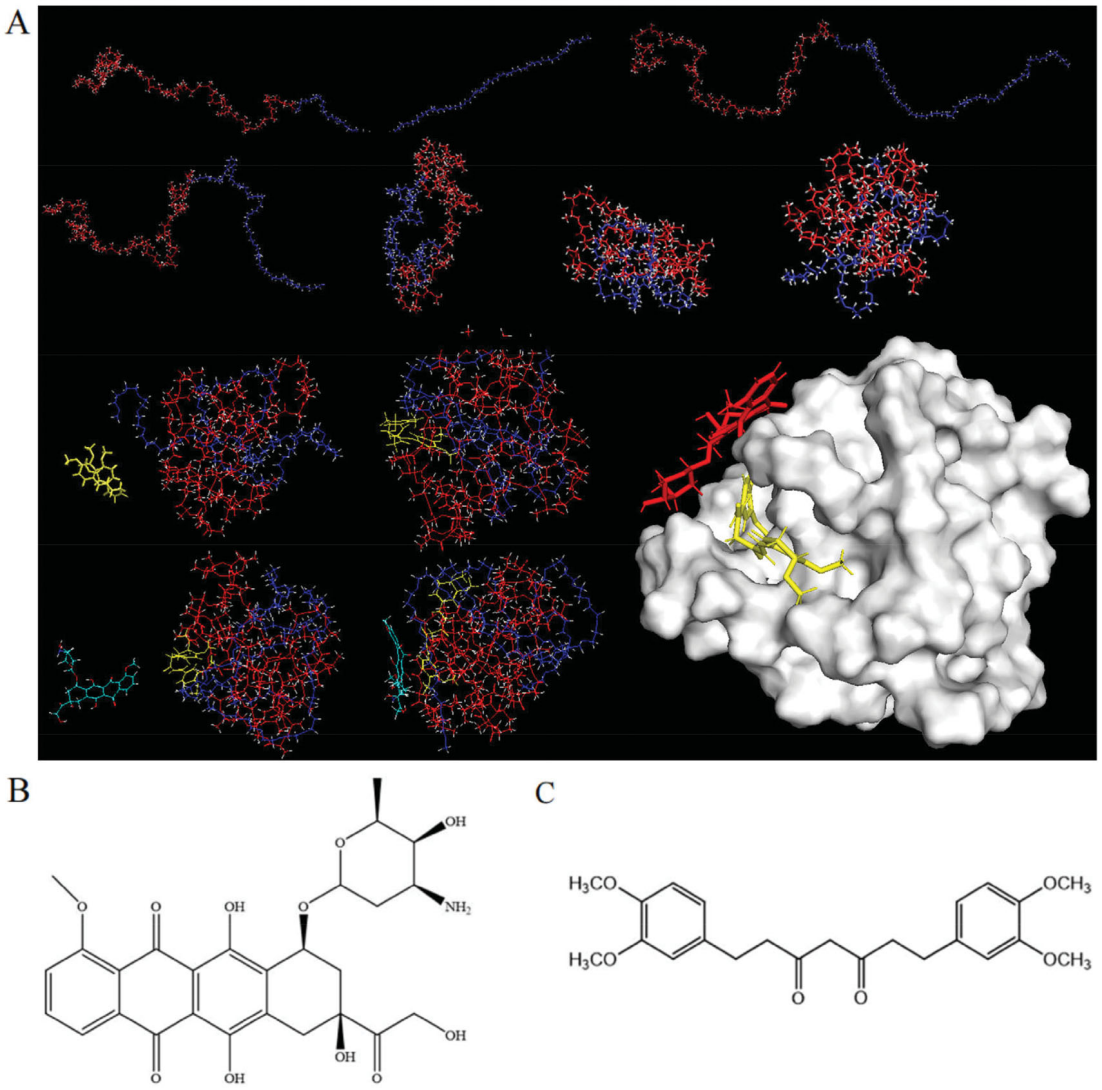
Molecular dynamic simulation illustrating (A) the self-assembly of copolymer mPEG-PLA, DOX (B), and DiMC (C) into the micellar system of CPM-DD. In part (A), the red line and blue-purple line represented mPEG and PLA, while the molecule in red was DOX, and that in yellow was DiMC, respectively.

In order to better understand the proposed mechanism of drug entrapment into CPM-DD, an *in silico* study was performed by using molecular dynamics (MD) and molecular mechanics (MM) methods, which made the theoretical structure of the randomly constructed polymeric carrier visualized for investigation of the molecular interactions among copolymer and small-molecule drugs DOX and DiMC. It was clearly demonstrated that the copolymer mPEG-PLA with an initial curve shape gradually bent and changed with the heating process. After 100 ps MD simulation, it eventually developed into a spherical shape containing a hydrophobic cavity and a hydrophilic shell, thus providing suitable sites for binding with small-molecule drugs. Meanwhile, the drugs periodically altered shape and distance from the copolymer to achieve superior interaction modes. In the end, DiMC was entrapped into the hydrophobic core of the spherical copolymer and DOX on the hydrophilic surface, respectively (Figure 4A).

The structure characteristics of the two small-molecule drugs may be responsible for such a particular mode of copolymer-drug interaction (Zhang et al., 2018). Chemically DOX is a kind of drug molecule with moderate hydrophilicity. The glycosidic group would contribute to its high water solubility and the preference for attaching to the hydrophilic surface of micelles (Figure 4B). In contrast, the lipophilic DiMC could be entrapped into the inner hydrophobic core of the micelles through strong hydrophobic interaction during amphiphilic self-assembly. Meanwhile, the acidic hydroxyl group in DiMC from keto-enol tautomerism (Sohail et al., 2021a; Jayakumar et al., 2016) could lead to efficient interaction with DOX via its basic amino group (Figure 4C). In view of this, DiMC could be regarded as a vital link that significantly improved the interactions among the copolymer and both drugs, therefore resulting in high encapsulation yields of CPM-DD, particularly a remarkable increase in the encapsulation efficiency of DOX. In fact, DOX alone could barely be encapsulated into micelles under the same conditions since no noticeable Tyndall effect was observed in this system (Figure 3A). The results demonstrated the unique molecular mechanism relating to the construction of CPM-DD, which would significantly contribute to specific characteristics of this complex micellar system, such as the distinct drug release profiles mentioned above (Figure 3F).

### 3.5. Cellular localization

**Figure 5.**
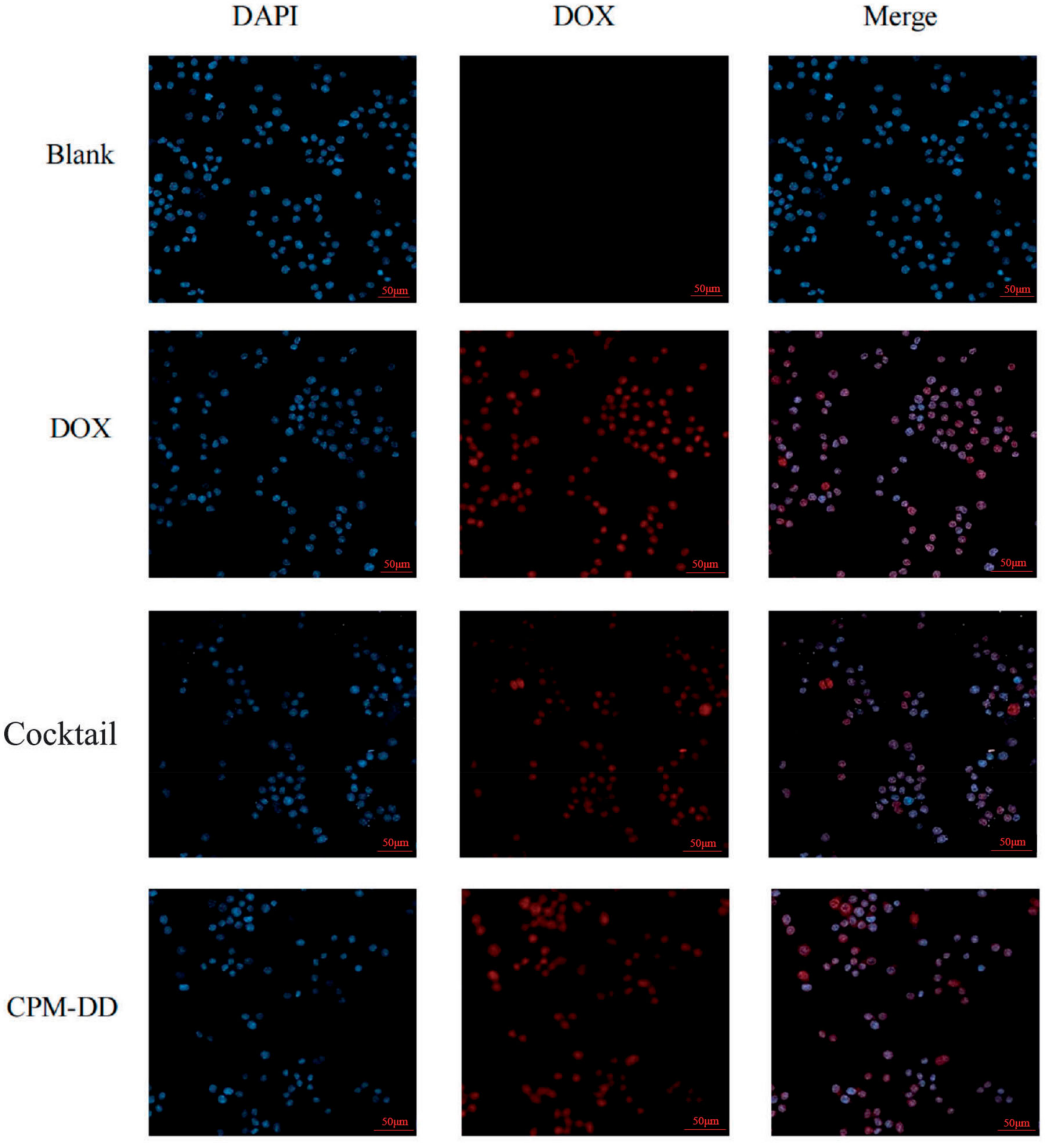
CLSM observation of *in vitro* cellular uptake of DOX by MCF-7 cells from various formulations such as the free drug solution (DOX), cocktail solution of DOX and DiMC (Cocktail), the complex micelles (CPM-DD), and the vehicle saline for control (Blank).

To better understand the intracellular drug release characteristics of the dual drug-loaded micellar system of CPM-DD, the cellular localization was further evaluated after an overall characterization. MCF-7 cells in the groups of CPM-DD, DOX, Cocktail, and Blank were treated with CPM-DD, the cocktail of DOX and DiMC, and free DOX alone for comparison, and blank micelles as control, respectively. After the cell nuclei were stained, CLSM observation was performed by detecting the blue fluorescence of DAPI and the obvious specific red fluorescence of DOX. Consequently, a continuous increase in the red fluorescence intensity could be observed by extending the incubation time. In contrast to the Blank group, all the other three groups treated with an equivalent amount of DOX obviously displayed strong red fluorescence after 2-h incubation. The images in the Merge column further illustrated that the regions with red fluorescence were well overlapped with those with blue fluorescence, and there was no noticeable difference among these DOX containing groups (Figure 5), suggesting that the biodegradable complex copolymer micelles of CPM-DD might efficiently release drugs into cancer cells without any influence on cellular uptake capacity.

DOX is a kind of anthracycline anti-cancer agent with the best-known mechanisms based on inhibition of DNA replication, transcription, and repair processes occurring in the nucleus, which thus is usually regarded as the final target location and the main target responsible for its anti-cancer potency (Sohail et al., 2021b). The results of the present study clearly indicated a rapid uptake of DOX by MCF-7 cells almost entirely into the nuclei, no matter what its preparation was. Also, it was confirmed that DOX could be well uptaken into tumor cells' nuclei, and CPM-DD would be an efficient nano-formulation of DOX with complete maintenance of the anti-cancer potency and the final target this chemotherapy drug as well.

### 3.6. *In vivo* responses of CPM-DD

Based on the female BALB/c nude mice bearing human breast cancer MCF-7 cells, *in vivo* responses of the CPM-DD preparation were investigated to better understand the systemic safety, anti-cancer efficacy, and bio-distribution characteristics as well. The cocktail formulation and free DOX alone under the same regime were used for comparison, while the mice only given the same volume of vehicle (saline) or blank micelles without any drug were used as controls.

**Figure 6.**
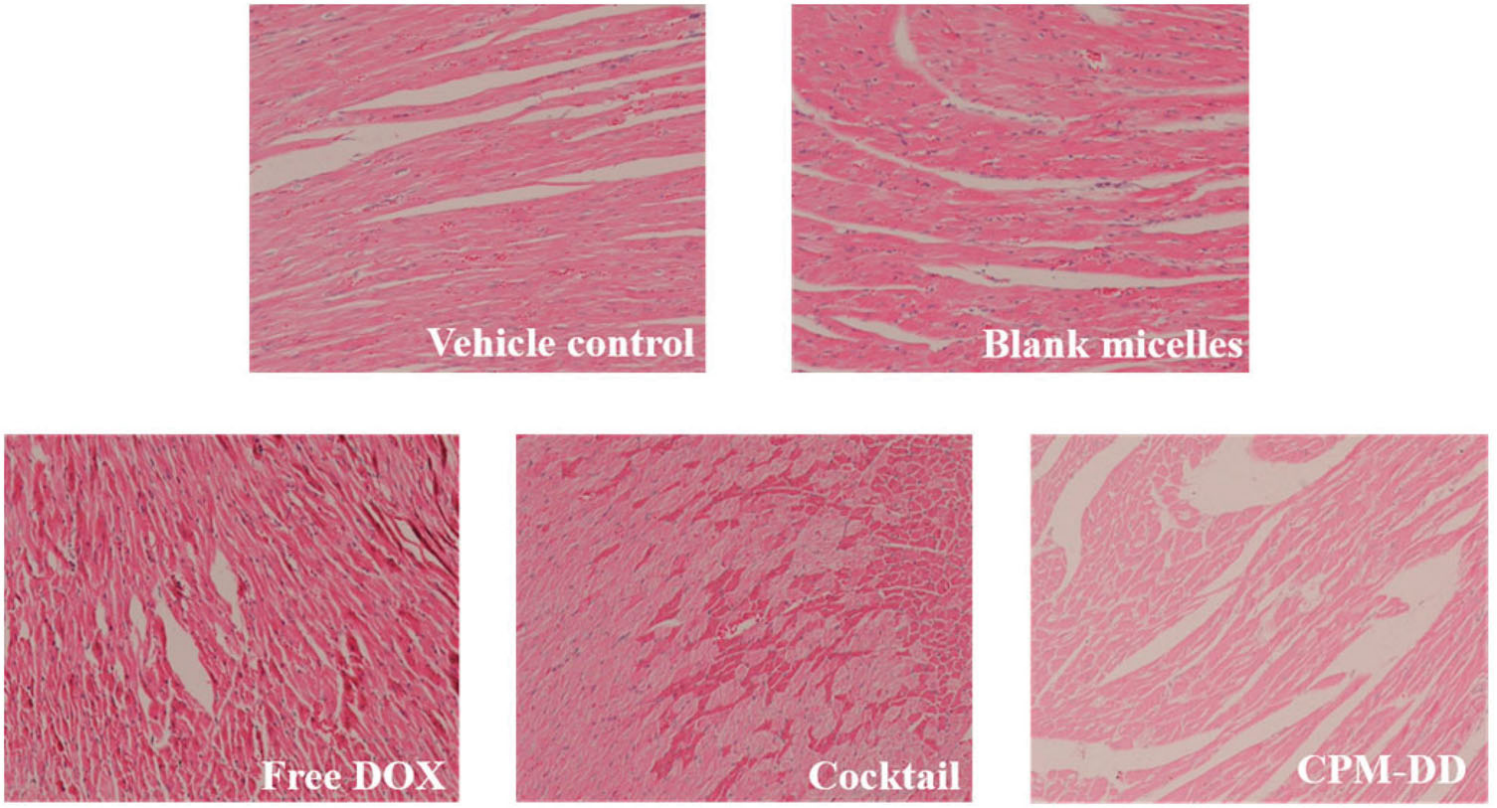
Evaluation of cardiotoxic effects in nude mice bearing MCF-7 cancer cells by light microscope observation (×400) of pathological change in mice left ventricles.

Taking into consideration the fact that cardiotoxicity is one of the major challenges of DOX-related chemotherapies (Sohail et al., 2021b; 2021c), the histopathological examination of cardiac tissue specimens was firstly performed to manifest the effect of CPM-DD against DOX-induced cardiac injuries. As illustrated in Figure 6, the tumor-bearing nude mice in the vehicle control group or blank micelles group showed basically normal morphology of cardiac myocytes in the left ventricle, while the DOX-containing treatments could affect cardiomyocytes in different ways and to different degrees depending on the formulation, among which the severest myocardial damage was observed in the mice only treated with free DOX. Accompanied by infiltration of inflammatory cells, the DOX alone group exhibited obvious myocardial injuries such as cross-striations, myocardial endochylema puffing and sarcoplasmic matrix partly resorbed, as well as myocardial fiber disarrangement, cellular swelling and degeneration, hinting toward toxin-mediated necrosis of cardiomyocytes. More to the point, these DOX-induced cardiac injuries could be alleviated by co-administering DiMC, especially through the CPM-DD micelles, which displayed less histopathological changes than the cocktail formulation at an equivalent dosage. These findings thus clearly demonstrated the high potency of DiMC against DOX-induced cardiotoxic effect by co-administering both drugs through the CPM-DD formulation.

**Figure 7.**
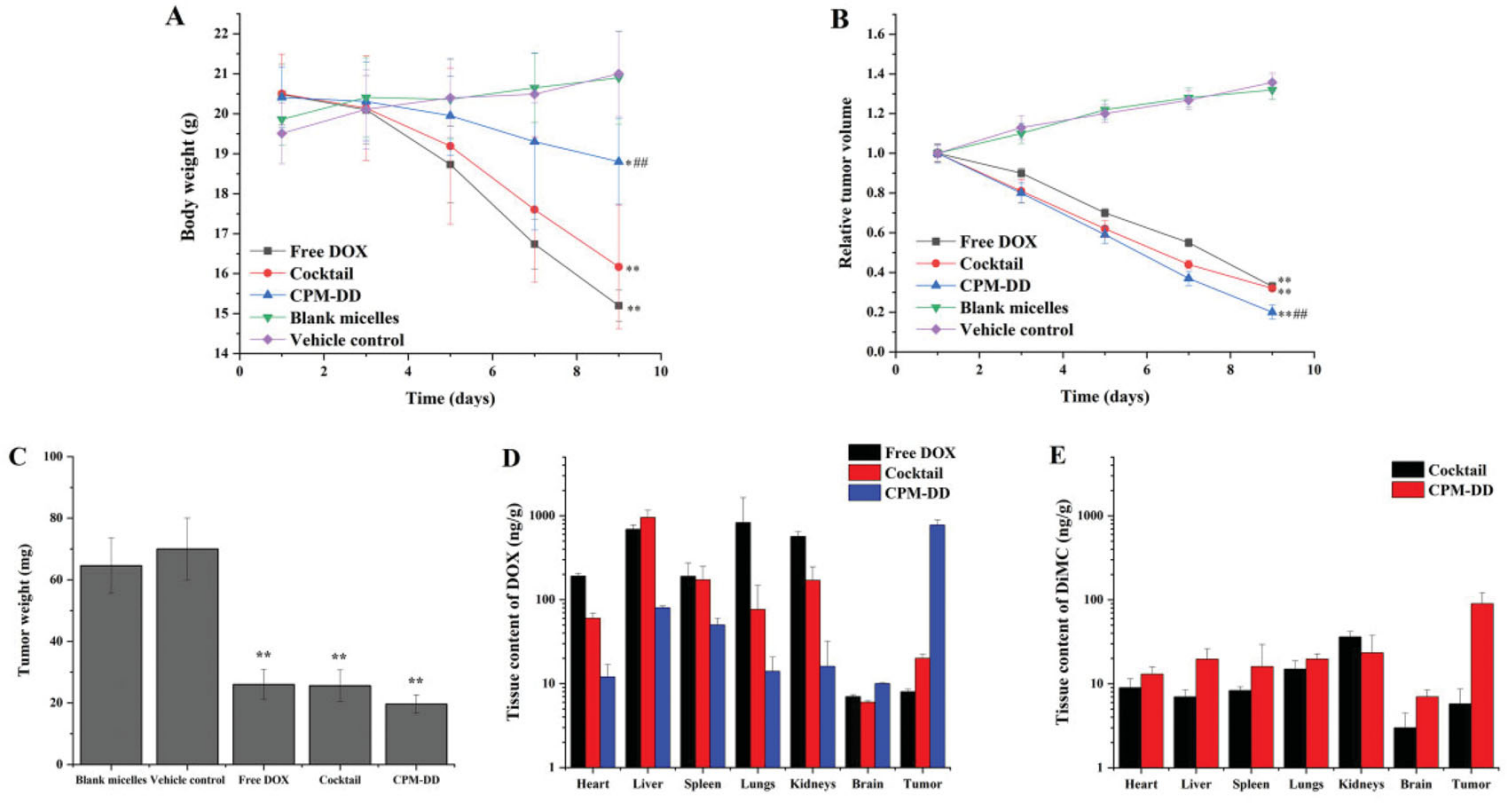
The *in vivo* responses of various formulations related to CPM-DD in MCF-7 breast tumor-bearing nude mice, including (A) body weight change, (B) relative tumor volume, (C) tumor weight, (D) tissue drug distribution of DOX, and (E) tissue drug distribution of DiMC after the last dosing. **p < .01 compared with blank controls, *p < .05 compared with blank controls, ##*p* < .01 compared with the free DOX group.

The systemic safety then was evaluated according to the results of body weight change during treatment. As shown in Figure 7A, the two control groups exhibited very similar patterns in the bodyweight change during administration (*p* > .05), and both had a slight increase (∼5–10%) after the last dosing, therefore indicating the safety of copolymer mPEG-PLA used for drugs loading in the present study. Meanwhile, significant differences could be observed among the three groups treated with DOX-containing drug formulations. However, they all showed a noticeable body weight loss at the end of the experiment. More to the point, the administration of free DOX alone or the cocktail formulation of DOX and DiMC led to nearly 20% weight loss, which was much greater than the other three groups (*p* < .01). Considering that weight loss might be due to the tumor burden, lack of energy, and the state of depression, these results thus demonstrated the systemic toxicity of free DOX in tumor-bearing mice, no matter whether it was administered alone or along with DiMC. It could also be concluded that the complex micellar formulation of CPM-DD would have a great potential to attenuate these DOX-related toxicities.

Tumor volume and tumor weight are the key factors for evaluating anti-tumor effectiveness. Herein the tumor volume was measured on a daily basis while the tumor from each mouse was weighed after the sacrifice at the end of the experiment. The relative tumor volume was further calculated by using the data of the first dosing as a reference. As shown in Figure 7B, the two control groups exhibited similar patterns in the tumor size change during administration, and both had an obvious increase (∼30–40%) after the last dosing. There was also no significant difference in tumor weight between blank micelles and the vehicle control group (*p* > .05, Figure 7C), suggesting that the copolymer mPEG-PLA used for drug loading would not directly affect the antitumor efficacy of DOX. When compared with the controls, the groups treated with DOX containing formulations (free DOX, the cocktail, or CPM-DD) all displayed similar patterns against *in vivo* tumor growth (*p* < .01), no matter what the formulation was. The complex micelles of CPM-DD could lead to nearly 80% tumor size loss after the last dosing, and even there was a significant difference with free DOX alone (*p* < .01). Altogether, these findings clearly demonstrated the significant advantages of CPM-DD versus the conventional formulations of DOX, such as free DOX solution and the simple cocktail of DOX and DiMC. In a word, CPM-DD would be a promising formulation of DOX for cancer therapy with attenuated toxicity and improved anti-tumor efficacy, which may benefit from the unique co-encapsulation of drugs into polymeric complex micelles.

In order to better understand the mechanism of action of CPM-DD, *in vivo* biodistribution analysis was performed for both drugs along with an evaluation of efficacy and toxicity in tumor-bearing nude mice, and the conventional formulations were used for comparison. The mice were sacrificed after the last dosing, then the tumor and the vital organs such as the heart, liver, spleen, lung, kidneys, and brain were harvested to determine drug content. Resultantly, these formulations indeed exhibited different drug delivery patterns. However, both drugs could be effectively distributed to most tissues in the mice administered with free DOX, the cocktail, or complex micelles. As shown in Figures 7D and E, CPM-DD was found to have much better drug localization in the tumor for both DOX and DiMC, but a much lower amount of DOX in cardiac tissues and other primary organs in contrast to the conventional formulations. DOX is very apt to induce cardiotoxicity, and its clinical application is restricted by the life-threatening cardiotoxic effects (Sohail et al., 2021b). Therefore, these results revealed the great advantages of CPM-DD in tissue drug distribution over conventional formulations of DOX.

It is well known that particle size is of the utmost importance for nano-delivery systems (Li et al., 2020). The suitable particle size may provide a high chance of avoiding macrophage engulfment and clearance by the reticuloendothelial system (RES) or mononuclear phagocyte system (MPS), which could also improve drug accumulation at the tumor site through enhanced permeability and retention effect (EPR). As mentioned above, the complex micelles of CPM-DD herein were uniform nanoparticles with a hydrophilic shell and a mean size of about 30 nm. Moreover, these particles exhibited good colloidal stability in blood circulation (Figure 3). Therefore it could be concluded that such a micellar structure was mainly responsible for the characteristic *in vivo* responses of the CPM-DD formulation, including the passive tumor targeting of both drugs that led to improved anti-cancer efficacy and minimal side effects on the main organs.

## 4. Conclusion

In the present study, a polymer-based nanomicellar system co-delivering DOX and DiMC, namely CPM-DD, was successfully developed to take advantage of combination therapy and overcome the challenges of conventional formulations of DOX for cancer therapy. The complex nanomicelles could be reliably obtained by self-assembly under optimal formulation and processing conditions. Systematic evaluation based on various models clearly demonstrated the characteristics of drug loading and drug release that were closely related to the improved anti-cancer potency and attenuated toxicity of CPM-DD, which, therefore, would be an excellent alternative to the conventional formulations of DOX and worthy of clinical application for cancer chemotherapy.
